# Homozygosity for a missense mutation in the 67 kDa isoform of glutamate decarboxylase in a family with autosomal recessive spastic cerebral palsy: parallels with Stiff-Person Syndrome and other movement disorders

**DOI:** 10.1186/1471-2377-4-20

**Published:** 2004-11-30

**Authors:** Clare N Lynex, Ian M Carr, Jack P Leek, Rajgopal Achuthan, Simon Mitchell, Eamonn R Maher, C Geoffrey Woods, David T Bonthon, Alex F Markham

**Affiliations:** 1 Molecular Medicine Unit, University of Leeds, Clinical Sciences Building, St James's University Hospital, Leeds, UK; 2 Neonatal Medical Unit, St Mary's Hospital, Manchester, UK; 3 Department of Paediatrics and Child Health, Section of Medical and Molecular Genetics, The Medical School, University of Birmingham, Birmingham, UK

## Abstract

**Background:**

Cerebral palsy (CP) is an heterogeneous group of neurological disorders of movement and/or posture, with an estimated incidence of 1 in 1000 live births. Non-progressive forms of symmetrical, spastic CP have been identified, which show a Mendelian autosomal recessive pattern of inheritance. We recently described the mapping of a recessive spastic CP locus to a 5 cM chromosomal region located at 2q24-31.1, in rare consanguineous families.

**Methods:**

Here we present data that refine this locus to a 0.5 cM region, flanked by the microsatellite markers D2S2345 and D2S326. The minimal region contains the candidate gene *GAD1*, which encodes a glutamate decarboxylase isoform (GAD_67_), involved in conversion of the amino acid and excitatory neurotransmitter glutamate to the inhibitory neurotransmitter γ-aminobutyric acid (GABA).

**Results:**

A novel amino acid mis-sense mutation in GAD_67 _was detected, which segregated with CP in affected individuals.

**Conclusions:**

This result is interesting because auto-antibodies to GAD_67 _and the more widely studied GAD_65 _homologue encoded by the *GAD2 *gene, are described in patients with Stiff-Person Syndrome (SPS), epilepsy, cerebellar ataxia and Batten disease. Further investigation seems merited of the possibility that variation in the *GAD1 *sequence, potentially affecting glutamate/GABA ratios, may underlie this form of spastic CP, given the presence of anti-GAD antibodies in SPS and the recognised excitotoxicity of glutamate in various contexts.

**Table 4:** *GAD1* single nucleotide substitutions detected on mutation analysis and occurring in sequences submitted to NCBI SNP database and in the literature. This is not a definitive list, but includes those described at the time of the mutational analysis. *Nucleotide positions were not provided by Maestrini et al. [47].

**Source**	**SNP position in mRNA, from the translational start site (bp)**	**Gene position of SNP(bp)**	**Amino acid change**
(A)Lappalainen *et al*. (2002)	A(-478)Del	Exon 0 (73)	*No substitution*
(B)Lappalainen *et al*. (2002)	G(-147)A	Exon 0 (404)	*No substitution*
(C)Lappalainen *et al*. (2002)	A(-39)C	Exon 1 (25)	*No substitution*
(D)Spastic CP patients family B	G(36)C	Exon 1 (97)	*Ser(12)Cys*
(E)NCBI collated resource	G(48)C	Exon 1 (104)	*Pro(17)Ala*
(F)Control samples & family A NCBI collated resource	T(110)C	Exon 2 (29)	*No substitution*
(G)Kure *et al*. (1998)	T(315)C	Exon 4 (14)	*No substitution*
(H)Bu and Tobin (1994) Kure *et al*. (1998)	A(407)G	Exon 4 (105)	*No substitution*
(I)Maestrini *et al*. (2002)*	G/C	Intron 4	*No substitution*
(J)NCBI collated resource	C(696)T	Exon 6 (56)	*No substitution*
(K)Lappalainen *et al*. (2002)	T/Del	Intron 7 (35)	*No substitution*
(L)In control samples Lappalainen *et al*. (2002)	T/C	Intron 8 (185)	*No substitution*
(M)Maestrini *et al*. (2002)*	C/T	Intron 9	*No substitution*

## Background

Cerebral palsy (CP) is a term used to define a group of disorders [1] characterized by a non-progressive abnormality of posture and movement, resulting from defects in the developing nervous system [2]. Approximately 1 in 250 to 1000 live births presents with CP, making it one the commonest congenital disabilities [3]. Many different aetiological factors have been implicated. Among preterm infants, the incidence of CP generally increases with decreasing gestational age and the origin in most cases may be traced to post/peri-partum periventricular leukomalacia and intraventricular/periventricular haemorrhage [4]. Conversely in term infants perinatal causes can only confidently be attributed where there is documented perinatal hypoxia/acidosis and clinical encephalopathy in the early neonatal period [5]. Prenatal risk factors in the aetiology of CP include low birth-weight, intrauterine infection and exposure to teratogens during pregnancy [6,7]. The cause in a large proportion of cases remains obscure.

Depending on the overall clinical picture, CP can be sub-classified into a number of phenotypic groups [8,9]. Dyskinetic CP accounts for ~20% of all cases, which may be further divided into choreoathetotic (5%) and dystonic (15%) forms. Ataxic CP (~10% of all cases) can also be sub-divided into two forms, simple (congenital) ataxia (5%) and ataxic diplegia (5%). Spastic CP is the most prevalent sub-type (~70%) and was the phenotype of the probands in this study [10]. It is characterised by muscular hypertonicity and pronounced rigidity of the affected limbs. Spastic CP can be sub-classified according to the topography of the affected limbs as hemiplegic (20%), monoplegic (<1%), diplegic (40%), or quadriplegic (10%) [11].

Kuban and Leviton [3] suggested that CP could be genetic in origin, as well as the result of environmental insult at any point during CNS development. Most estimates place the proportion of CP cases with a genetic aetiology at between one and two percent of the total [12]. Among infants and children with spasticity, symmetry of neurological signs has been identified as a strong indicator of a probable genetic aetiology [13,14]. The proportion of cases demonstrating Mendelian inheritance varies among the different sub-types of CP [2,13,7]. X-linked, autosomal dominant and recessive inheritance patterns have been described for non-progressive CP. An ataxic diplegic autosomal recessive trait (OMIM:605388) [15] has been mapped to chromosome 9p12-q12. Progressive spastic paraplegia (SPG) has a similar pathology to CP. SPG displays autosomal dominant (SPG3A at 14q11-q21 encoding the atlastin GTPase; SPG4 at 2p21-22 encoding spastin, an AAA family ATPase/chaperonin; SPG6 at 15q11.1; SPG8 at 8q23-q24; SPG9 at 10q23-q24; SPG10 at 12q13; SPG12 at 19q13; and SPG13 at 2q24 encoding the HSP60 mitochondrial chaperonin), recessive (SPG5A at 8cen; SPG7 at 16q24.3 encoding paraplegin, an AAA family ATPase/inner mitochondrial membrane chaperonin; SPG11 at 15q13-q15; SPG14 at 3q27-q28; SPG15 at 14q22-q24; and SPG17 at 11q12-q14), or X-linked inheritance patterns (SPG1 at Xq28 encoding the L1CAM adhesion molecule; SPG2 at Xq22 encoding proteolipid protein-1; and SPG16 at Xq11.2).

A non-progressive, autosomal recessive, symmetrical spastic CP locus has been mapped to a 5 cM region between D2S124 and D2S333, at 2q24-31.1 (LOD score of 5.75) in consanguineous families originating from the Mirpur region of Pakistan (OMIM:603513) [10]. Affected individuals had no identifiable perinatal cause of CP, or underlying diagnosis and presented with developmental delay, mental retardation and sometimes epilepsy as part of the phenotype. We initially performed detailed physical mapping of the 5 cM region, so as to accurately define the marker order and to refine the linkage interval. The positions of a large number of genes and ESTs were defined accordingly, allowing the rapid identification of candidate disease genes. The minimum region of homozygosity was reduced to 0.5 cM by typing large numbers of microsatellite markers in the families. Subsequently, portions of this region have been sequenced in the human genome project. Within the region we have concentrated on the positional candidate *GAD1*, which codes for the 67 kDa isoform of L-glutamate decarboxylase (GAD EC:4.1.1.15).

GAD requires the cofactor pyridoxal 5'-phosphate (PLP) and catalyses the production of Gamma-aminobutyric acid (GABA) from glutamate [16]. Two separate, independently-regulated genes, *GAD1 *and *GAD2 *(at chromosome 10p11, encoding a 65 kDa GAD isoform), have presumably arisen by duplication and been conserved during evolution, as indicated by their sequence homology [17] and the retention of common intron-exon boundary splice sites [18]. Their *N-*termini demonstrate ~23% homology, but their *C-*termini, which contain the catalytic site, have ~73% amino acid sequence identity between the isoforms [19]. GABA and glutamate are the most abundant amino acid neurotransmitters in the brain. GABA, an inhibitory neurotransmitter, and excitatory glutamate, both play important roles in synaptic plasticity and neuroendocrine function [20]. Both isoforms of GAD are also involved in intermediary metabolism, participating in the GABA shunt, which bypasses two steps of the TCA cycle [21]. GAD activity of both isoforms, is ubiquitous, but highest in the brain and pancreatic islets of Langerhans. We therefore performed detailed mutational screening of *GAD1 *in familial spastic CP probands and unaffected family members.

## Methods

### Features and pedigrees of ascertained families

#### *Family A *(4718/4719)

The oldest affected male diagnosed with non-progressive, spastic CP, demonstrated global developmental delay, with no associated neurological abnormalities and moderate mental retardation. His affected younger sister was also diagnosed with spastic CP, global developmental delay and moderate mental retardation.

#### *Family B *(4578/4579/4581/4679)

The oldest non-progressive, spastic CP female has severe mental retardation, and is confined to a wheelchair. The next oldest spastic CP male has severe mental retardation, mild hypertonia and ataxia of the upper limbs. The next oldest spastic CP male is a dizygotic twin born by Caesarean section. This patient on presentation demonstrated developmental delay, mild hypertonia and ataxia of the upper limbs. The youngest affected female is not able to walk or stand unaided and has severe developmental delay. Details of the clinical picture in these pedigrees have been described previously [10,14].

### Physical mapping of candidate region

ICI and CEPH YAC libraries were screened by PCR amplification of STSs that were mapped between D2S2157 and D2S385. The positive clones (CEPH human mega YAC clones: 761-G10, 797-G4, 842-G1, 842-G3, 910-G12, 945-C12; ICI human YAC clones: 13I-E10, 13I-G11, 14I-G12, 16F-H2, 1E-F6, 21E-G5, 30A-D10, 30H-D2, 33D-C4, 35B-D2, 18B-E3, 31H-A4, 40D-E8, 8D-E12, 9H-F10) were obtained from the UK HGMP Resource Centre . Clones were grown up in casamino acid selective broth overnight and harvested by centrifugation. The pellet was then washed twice in 0.5 ml 100 mM Tris-HCl, pH7.5, 0.5 M EDTA buffer. After a second round of centrifugation the pellet was resuspended in molten 1% LMP agarose in 5 mM Tris-HCl, pH7.5, 0.05 M EDTA, 10 mM NaCl with 100 μg of Zymolase. The agarose was cooled and the resultant plugs were incubated overnight at 37°C in 50 ml of 0.5 M EDTA, 10 mM Tris-HCl, pH7.5, 10 mM NaCl. The buffer was replaced with fresh solution to which 100 μl of 40% Sarkosyl NL30 and 50 μl Proteinase K (1 mg/ml) was added and the plugs were incubated overnight at 50°C.

The YAC chromosomal DNA was purified and separated for sizing by CHEF electrophoresis. The switching angle was 120°, using a CHEF™ electrophoresis tank (Bio Rad), run for 16 hours at 6 V/cm, 10°C with an initial pulse time of 30 sec to a final pulse time of 90 sec. DNA was stained with (20 mg/ml) ethidium bromide solution (BDH) for 2 hours and visualized under ultra-violet illumination. Southern blotting and membrane hybridisation of CHEF gels were performed on Hybond-N™ membranes (Amersham). DNA was immobilised by heating the membrane to 80°C in vacuum for 1 hour in a gel dryer (Bio-Rad). Radio-labelled YAC vector-specific probes were generated using the Megaprime random primer kit (Amersham) as described in the manufacturer's instructions, using PCR products as a template. Sizes were estimated based on comparisons with the known sizes of the native yeast chromosomes.

### Genetic mapping

The microsatellite markers: Cen-D2157, D2S124, D2S2330, CHLC.GATA71B02 (D2S1776), D2S2345, CHLC.GATA71D01, D2S294, AFMA109YC1, D2S376, D2S2284, D2S2177, D2S2194, D2S333, D2S2302, D2S2381, D2S326, AFMA304WB1, D2S138, D2S148, D2S300-Tel, were amplified using fluorescently labelled primers (Lifetech) previously designed by the Whitehead Institute or on the GDB database , , . These primers were used to amplify microsatellite marker alleles from individuals by PCR. Individual alleles were identified by denaturing polyacrylamide gel electrophoresis on an ABI Prism 377 sequencer and analysed using Genescan™ and Genotyper™ (version 1.1.1) software (Applied Biosystems).

### Single-strand conformational polymorphism analysis

*GAD1 *exon sequences were amplified by PCR using the primers described in Table 1. SSCP was performed on GeneGel Excel (Amersham), using a 12.5/24 gel (14°C, 600 V, 25 mA, 15 W for 80 min) and the DNA was visualised by silver staining as per the manufacturer's instructions.

**Table 1:** Oligonucleotide primer used in the amplification of GAD1 exons.

**Primer**	**Forward**	**Reverse**
*Exon 1*	dGCCCCATTTATTTCCCAGCC	dGCACAGCTCTCGCTTCTCTT
*Exon 2*	dGAAAACCATTGTCCTCCACC	dGCCTGTCGGCTCACAGATT
*Exon 3*	dACCAGCTTCTTGTGCCATAG	dATCTACTGGCTAGCATGGGG
*Exon 4*	dATTCCATGTCTGAGCAGCCT	dACTGTTACTGCCCAAGCTTG
*Exon 5*	dGCCGTTTGCCTTCAAGATAG	dAGAACCACTGGGACTGAACT
*Exon E*	dACCAGTATCTCCTCGCCATG	dTTGGGAGGCCCCTGGAAATT
*Exon 6*	dACCCAACTACAAATACTAAACC	dAATAGGAAGTCAGGGTATCC
*Exon 7*	dGAGACACCAGCTCAGCGTTC	dCTGCAACAAACAGAGGCTCG
*Exon 8*	dGTCGGGGATGCTTTCTCCATG	dCTCAGTACATTGTGCCAAGC
*Exon 9*	dCAAGCTGCTAATGGTCTGTT	dGTCTCATATTATCAAGGACTG
*Exon 10*	dCACAATTCTTCTTCCTGTGA	dTGGGGAGGAGCTTGAGGCAA
*Exon 11*	dACAATCAGTGTGGGCTGAAC	dGAAGCAAACTTAGACCGAAA
*Exon 12*	dCTTGAGTTGGAATGGGTGTT	dACTGCAAAGAGACCCCACGT
*Exon 13*	dTCCTTCCAAGCAGCCTAGTT	dGTGATATATCTTTGCCCCTC
*Exon 14*	dGACAGCATAGCCTTCCCAAA	dCATGTTGCCAGAAGCTTCAG
*Exon 15*	dGGTTTGGGAACAGCTTTCTC	dTTCCCCCACTAGAAAGGCAC
*Exon 16*	dGTTAAAAAGAGAGGGTGTTC	dCCCTCAATGAAATGGCCTGT

### Sequencing of GAD1 exon sequences

Primers used to amplify the exons of *GAD1 *were designed from the sequence of BAC RP11-570c16 and obtained from Lifetech (Table 1). PCR products were purified from agarose gels using QIAquick gel extraction kits (Quiagen), sequenced using ABI PRISM Big Dye Terminator Cycle Sequencing Ready Reaction Kits (Applied Biosystems) and then analysed using an ABI Prism 377 automated sequencer.

## Results

### Physical map of the spastic CP locus

**Figure 1:**
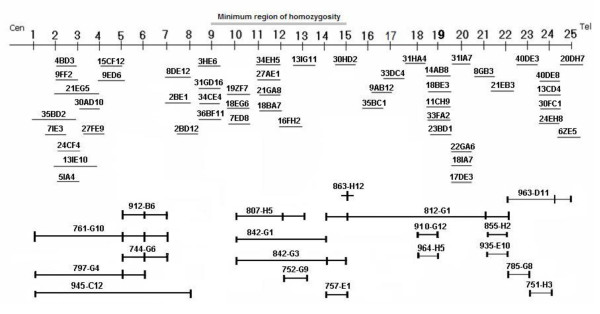
***An integrated physical YAC contig spanning the human chromosome 2 spastic CP locus. ***This was constructed against a framework of microsatellite and STS markers, to incorporate the region of linkage identified by genotyping data. The positions of microsatellite and STS markers are represented numerically left to right from centromere to telomere. These loci 1–25 were mapped arbitrarily equi-distant onto the contig, in the following order: Cen-(1) D2S2157; (2) D2S382; (3) WI-18792; (4) D2S124; (5) D2S111; (6) D2S2384; (7) D2S2330; (8) D2S399; (9) D2S2345; (10) D2S294; (11) D2S2188; (12) D2S2284; (13) D2S2177; (14) D2S335; (15) D2S326; (16) D2S2381; (17) D2S2302; (18) D2S2307; (19) D2S2257; (20) D2S2314; (21) D2S138; (22) D2S148; (23) D2S2173; (24) D2S300; (25) D2S385-Tel.

An integrated YAC (ICI and CEPH mega YAC libraries) and RP-9 PAC (HGMP Resource Centre) contig for the interval D2S2157 to D2S385 was constructed (Figure 1). This map provided physical continuity connecting 25 loci from centromere to telomere spanning the entire 2q24.3-31.1 cytogenetic band region. These data were combined with BAC contigs, constructed at Washington University , in an attempt to form an ordered BAC/YAC contig across the minimum region of interest. Selected YACs from this contig were sized (Table 2) enabling the physical length of the region to be estimated. The partial contig map included 3 identified CEPH mega-YAC contigs (contig 1: 945-C12; 912-B6; 744-G6; 797-G4; 761-G10; contig 2: 752-G9; 757-E1; 807-H5; 842-G1; 842-G3 and contig 3: 964-H5; 935-E10; 855-H2; 785-G8; 963-D11; 751-H3). The size of the region incorporating the three contigs was estimated to be at least 2870 kb. The sizes of the ICI YACs were estimated to encompass a minimum locus of 2940 kb. Microsatellite markers, ESTs and known genes, mapped to this region by NCBI, were then located on the physical contig by PCR and BLAST sequence homology searches of the Washington University BAC contigs. The expression profiles of unidentified EST clusters were determined and used to form EST "bins". These contained groups of ESTs, which mapped to adjacent locations and showed common expression profiles, suggesting that they might represent different exons of the same gene. This placement of known genes and ESTs onto the physical map provided an annotation of the gene content of the region, at the time was constructed before any such facility was available from the HGP, as the focus for candidate disease gene selection.

**Table 2:** Approximate sizes of YAC clones spanning the 2q24-31.1 autosomal recessive spastic CP disease gene locus, used to estimate the minimum physical size of the region (kb). The CEPH MEGA and ICI YACs were sized using CHEF PFGE compared against the native yeast chromosomes. This confirmed the estimated size ranges of the YAC inserts, predicted by the Whitehead Institute (WI) database of YAC clones.

**CEPH human MEGA-YAC clones**		**ICI human YAC clones**	
***Clone***	***Size (kb)***	***Clone***	***Size (kb)***

910-G12	1630	40D-E3	290
744-G6	1120	35B-D2	260
912-B6	1190	33D-C4	120, 480
945-C12	1540	30H-D2	250
842-G3	1330	30A-D10	260
797-G4	1000	18B-E3	120
807-H5	1680, 1290	16F-H2	220
752-G9	1740	13I-G11	230
785-G8	690, 1060	13I-E10	460
842-G1	1380	8D-E12	280
757-E1	1100		
812-G1	1540		
863-H12	1550		
935-E10	1360		
785-G8	1060		
751-H3	1780		
963-D11	1670, 890		

### Genetic mapping data

**Figure 2:**
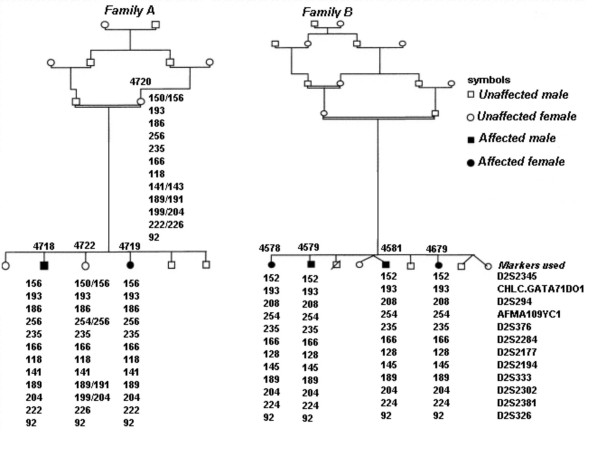
***Annotation of two pedigrees of spastic autosomal recessive CP families and corresponding linkage mapping data***. The markers shown are those that demonstrate the minimal homozygous region between the affected individuals of both families.

From the PAC/YAC contig, 20 polymorphic microsatellite markers were identified that span the chromosome 2q24-31.1 CP critical region. These were then used, with informed consent and local research ethics committee approval, in the detailed mapping of previously linked families [10] (Figure 2). These data did not support the presence of a founder mutation for autosomal recessive spastic CP, in that families did not share a common haplotype across the minimal homozygous region between D2S2345 and D2S326.

### Sequence analysis of GAD1

**Figure 3:**
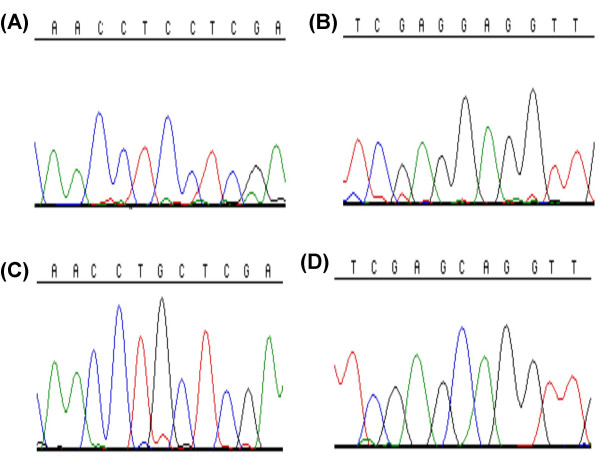
***Electropherograms of the sequence of the exon 1 SNP of GAD1 identified in the process of mutational analysis***. (A) and (B) show the normal C variant in the forward and reverse directions, respectively. (C) and (D) show the alternative G variant in the forward and reverse directions, respectively. This variant was only found in affected individuals of family B. No heterozygous individuals were identified for this nucleotide variant.

**Figure 4:**
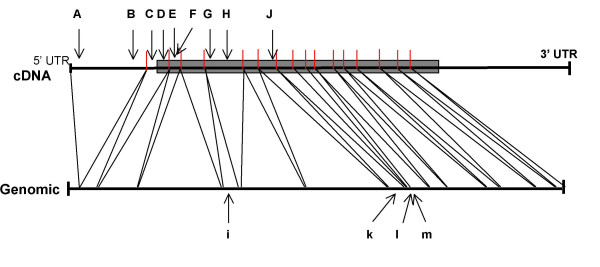
***An annotation of the distribution of single nucleotide substitutions identified in the open reading frame of GAD1. ***The approximate positions with respect to intron-exon of the open reading frame structure are illustrated. These were determined by sequencing of the probands in this study, from published data and from the NCBI collated database of SNPs. The letters refer to the SNPs listed in Table 4. Upper case letters refer to SNPs in the cDNA and lower case letters indicate SNPs in the genomic DNA. **A**: G(36)C, **B**: G(210)A, **C**: G(253)C, **D**: T(315)C, **E**: A(407)G, **F**: C(696)T, **G**: C(1506)T, **H**: C(1575)T, **i**: T(1625)G, **J**: C(1654)T, **k**: A(1659)G, **l**: G(1799)A, **m**: C(1899)A.

*GAD1 *was sequenced in affected and unaffected individuals in both families ascertained. In order to differentiate possible disease-causing mutations from polymorphisms, 100 control individuals were screened by SSCP to detect any *GAD1 *sequence variations in the normal population. SSCP variants were then sequenced to identify the underlying nucleotide substitutions. An homozygous G(36)C (Figure 3A,3B) nucleotide change was observed in 4 affected patients, which generated a Ser(12)Cys amino acid substitution. No obligate carriers were identified for this mutation. This variant has not been previously described and was not present in 200 normal chromosomes. A number of other sequence changes were detected, but none of these resulted in amino acid changes. These variants and all those recorded previously in the literature are presented in Table 4 and Figure 4. For those rare variants in the databases, which result in amino acid changes, no homozygous or compound heterozygous individuals have yet been described.

**Table 3:** Autozygosity mapping data generated by genotyping eight members of the two autosomal recessive spastic CP families. Subjects 4718, 4719, 4720 and 4722 represent family A; subjects 4578, 4579, 4581 and 4679 represent family B as demonstrated in Figure 2. This Table is organised according to the definitive marker order determined from current databases and the physical contig mapping undertaken. The minimum homozygous region is highlighted. *Denotes an unaffected family member.

**Marker**	**Base position**	**4718**	**4719**	4722*	4720*	**4578**	**4579**	**4581**	**4679**
D2S2157 AFMA119YH5	166029867	145	145	145/147	145	145/147	145/147	145/147	145/147
D2S124 AFM094ZC9	166347755	160	160	160	160	160/163	160/163	160/163	160/163
D2S2330 AFMC015YD9	166900134	156	156	156/158	156/158	160/168	160/168	160/168	160/168
CHLC.GATA71B02	167624503	240/256	240/256	240/256	240/256	240/256	240/256	240/256	240/256
D2S2345 AFM080XG9	168922932	**156**	**156**	**150/156**	**150/156**	**152**	**152**	**152**	**152**
CHLC.GATA71D01	169848018	**193**	**193**	**193**	**193**	**193**	**193**	**193**	**193**
D2S294 AFM205XF12	170579380	**186**	**186**	**186**	**186**	**208**	**208**	**208**	**208**
AFMA109YC1	171576976	**256**	**256**	**254/256**	**256**	**254**	**254**	**254**	**254**
D2S376 AFM319XG1	171576985	**235**	**235**	**235**	**235**	**235**	**235**	**235**	**235**
D2S2284 AFMB314YE1	171696071	**166**	**166**	**166**	**166**	**166**	**166**	**166**	**166**
D2S2177 AFMA155TF9	171790203	**118**	**118**	**118**	**118**	**128**	**128**	**128**	**128**
D2S2194 AFMA222XB9	171884139	**141**	**141**	**141**	**141/143**	**145**	**145**	**145**	**145**
D2S333 AFM2702E9	172592103	**189**	**189**	**189/191**	**189/191**	**189**	**189**	**189**	**189**
D2S2302 AFMB342ZD9	172758604	**204**	**204**	**199/204**	**199/204**	**204**	**204**	**204**	**204**
D2S2381 AFMA082TF5	172861090	**222**	**222**	**226**	**222/226**	**224**	**224**	**224**	**224**
D2S326 AFM266VE1	173299492	**92**	**92**	**92**	**92**	**92**	**92**	**92**	**92**
AFMA304WB1	176057526	130/132	122/132	130/132	122/132	124	124	124	124
D2S138 AFM176XD4	177947395	108	108	113	108/113	111/117	111	111	111
D2S148 AFM200WA11	178434054	184/188	184/188	184/194	184/194	188/190	186	186	186
D2S300 AFM214XC3	178826338	87/89	87/89	87/89	87/89	87/89	89	89	89

## Discussion

CP is a term used as a collective definition for a group of neurological disorders [3]. The pathophysiology in most cases is poorly understood, but includes genetic syndromes, congenital malformation, infective intra-uterine encephalitis, cerebral haemorrhage or infarction, ischemic damage, periventricular leukomalacia (PVL), and non-infarctive telencephalic leukomalacia [11]. The contribution of Mendelian inherited cases of CP accounts for approximately 2% of the total number [2,22]. A non-progressive form of autosomal recessive spastic CP has been identified [13]. McHale *et al*. [10] succeeded in identifying a 5 cM region on chromosome 2q24-31.1, which segregated with disease in consanguineous families. Linkage analysis identified a locus between markers D2S124 and D2S333, which produced a LOD score of 5.75, sufficient to warrant the further investigation described herein.

To refine and confirm the genetic marker order across a region, which was incompletely sequenced at the time, we used YAC and PAC clones to construct a physical framework and performed PCR to map ESTs and microsatellite markers to clones within the partial contig (Figure 1). When this contig was integrated with the BAC sequence contigs, rearrangement of the BAC order was necessary. With each subsequent DNA sequence update, the degree of inconsistency was reduced and this led to revision of microsatellite order compared with that used previously [10]. Using the YAC sizes, the sizes of gaps in the BAC contig could be estimated. Having generated a detailed map spanning the region, we selected microsatellite markers at evenly spaced intervals across the locus. These markers were used to refine the minimum region homozygous by descent in linked families, to between the markers D2S2345 and D2S326. The physical size of the region between these markers is approximately 0.5 cM. There was no suggestion of a founder haplotype common to the two families (Figure 2).

The Goldenpath Human Genome Working Draft Assembly 2001, is an annotation of the Washington BAC contig, combining sequence data of BACs, ESTs, known genes and hypothetical genes. We mapped ESTs and known, uncharacterised or hypothetical genes on the basis of sequence homology (NCBI , Whitehead  and Goldenpath databases), onto our YAC/PAC contig. ESTs were then collated according to their expression profiles to produce a "binned" EST map, on which candidate gene selection could be based. This reduced the number of hypothetical genes in the region and allowed the combination of genetic, physical mapping and expression data, into a single comprehensive map.

One interesting candidate within the minimal region was *GAD1*, which encodes GAD_67_. Expression of its transcript is ubiquitous, including the CNS. The main function of GAD_67 _is to catalyze the conversion of the excitatory amino acid and neurotransmitter glutamate to GABA, the main inhibitory neurotransmitter in the CNS [23]. In the developing CNS, GABA has an important role in neuronal differentiation and the control of plasticity [21]. GABA has also been implicated in the pathogenesis of various seizure and movement disorders [20].

**Figure 5:**
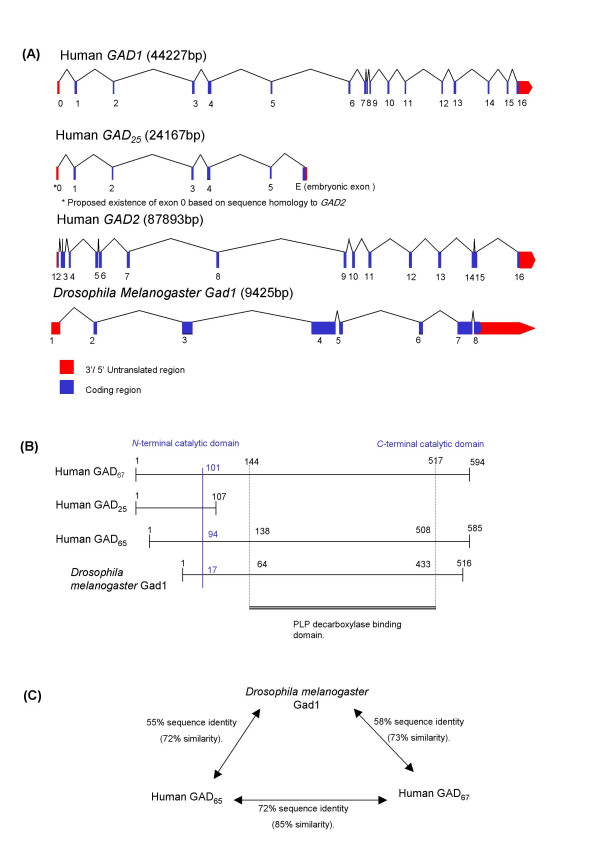
***Three illustrations of the genomic, protein and comparative sequence homologies of the different species of GAD. ***(A) The genomic structures of *GAD1*/*GAD_25_*/*GAD2 *and *Drosophila Gad1*. (B) Comparative protein domain structures of GAD_65_/GAD_25_/GAD_67 _and *Drosophila *Gad1. (Numbers represent approximate amino acid residues). (C) Schematic illustrating the relative homology of the protein structures of GAD_67_/GAD_65 _and *Drosophila *Gad1.

Vertebrates have two separate genes coding for GAD, which produce distinct forms of the enzyme. *GAD1 *and *GAD2 *have diverged relatively recently in evolution, as indicated by their degree of sequence homology and the retention of common intron-exon boundary splice sites [17] (Figure 5A). The variants of GAD differ in molecular weight, cellular and sub-cellular localisation, and their interaction with the cofactor PLP [18,20,24].

*GAD2*, located at 10p13-p11.2, is transcribed to produce a 5.6 kb mRNA in islets and brain, encoding a 65 kDa protein (585 AA residues). The 67 kDa (594 AA residues) form [17] is localised to 2q25-26 and encoded by a 3.7 kb transcript (*GAD1*) [23]. There is also evidence for a 25 kDa inactive protein (GAD_25_) produced from an alternatively spliced *GAD1 *transcript of 2 kb that contains an in-frame stop codon. This *GAD1 *splice variant has only been found in human islets, testis and adrenal cortex, although the homologue is present in fetal mouse brain [25]. GAD_67 _and GAD_65 _consist of two major sequence domains (Figure 5B). The *N*-termini (AA residues 1–94 in GAD_65 _and 1–101 in GAD_67_) demonstrate ~23% homology. These *N*-terminal domains are thought to be responsible for sub-cellular targeting and the formation of GAD_65_–GAD_67 _heterodimers [26]. The *C*-terminal domains (AA residues 96–585 in GAD_65 _and 102–594 in GAD_67_) contain the catalytic site, with ~73% sequence identity between the isoforms [19] (Figure 5).

In the CNS, GAD_65 _appears to be preferentially distributed in axon terminals and the associated synaptic vesicles, whereas GAD_67 _is also located in the cell bodies and more uniformly distributed throughout the neuron [24]. This suggests that each GAD isoform is involved in the synthesis of GABA in different sub-cellular compartments [21]. This is supported by the discovery that GAD_65 _is the main source of apoGAD (an inactive reservoir), which responds to short-term changes in neuronal activity and is more responsive to levels of PLP [18]. On the other hand, GAD_67 _predominantly exists bound to the PLP cofactor (holoGAD), providing a constitutive level of GABA production [20]. Bond *et al*. [27] showed that GAD_25 _is expressed in a temporally controlled manner, in the developing striatum and cortex in rodents, suggesting this may provide a mechanism of regulating GABA production in differentiating neurons.

Asada *et al*. [28] undertook the selective elimination of each GAD isoform in order to determine their respective roles. *Gad2*-/- mice are slightly more susceptible to seizures, consistent with an excitatory increase in the relative ratio of glutamate/GABA. However, they showed no obvious overall change in neuronal GABA content. Therefore GAD_67 _alone appears to produce sufficient GABA for effective neurotransmission [21]. *Gad1-/- *mice demonstrated a decrease of ~20% in total glutamate decarboxylase activity at birth. This was assayed by the conversion of ^14^C-labelled glutamate to ^14^CO_2 _in the presence of PLP. There was also a marked (7%) reduction in total GABA content in cerebral cortex homogenate measured by liquid chromatography [28]. Unfortunately, these mice died neonatally of severe cleft palate, masking any potential neurological dysfunction and also illustrating a role for Gad_67 _in non-neural tissues [21]. It is of interest that mice with mutations in the β-3 GABA receptor (GABRB3) at the Angelman syndrome (OMIM:105830) locus, also display cleft palate, implying a key role for GABA signalling in normal palate development [29,30].

Pyridoxine-dependent epilepsy (PDE) is a rare autosomal recessive disorder (OMIM:266100), characterized by generalized seizures during the first hours of life. The associated pathology may result from an alteration in the binding of the co-factor PLP to GAD. Interestingly epilepsy is commonly associated with CP and grand mal epilepsy developed at age six months in the two linked pedigrees described here [10]. *GAD1 *mutation was previously suspected of being the cause of PDE. Linkage of pyridoxine-dependent epilepsy has however been reported to 5q31.2-31.3, with *GAD1 *and *GAD2 *excluded [31]. Decreased levels of brain and CSF GABA, increased levels of CSF and cortical glutamate, and decreased levels of PLP in the frontal cortex, have been described in this condition.

GAD_65 _and GAD_67 _have been identified as auto-antigens in "Stiff Person Syndrome" (SPS, OMIM:184850), and in cerebellar ataxia [32-34]. GABA-mediated synaptic transmission is thought to be functionally impaired by the production of autoantibodies to GAD_65 _and GAD_67 _[35-37]. This results in a reduction in brain levels of GABA, prominent in the motor cortex, which can be demonstrated by Magnetic Resonance Imaging (MRI) in SPS patients. SPS is a disabling disorder characterised by muscle rigidity and episodic spasms of the musculature, thought to be due to autoimmune-mediated dysfunction of supraspinal GABAergic inhibitory neurons [38]. Hyperexcitability of the motor cortex in SPS has been demonstrated by transcranial magnetic stimulation [39].

Anti-GAD_65 _auto-antibodies in the CSF of ataxic and SPS patients selectively suppress GABA-mediated transmission in cerebellar Purkinje cells, without affecting glutamate-mediated transmission [37,40]. Low CSF levels of GABA have been reported in patients with Kok disease (OMIM:149400 also known as hyperexplexia/exaggerated startle reaction/startle disease) [41]. The exact mechanism by which autoantibodies target these intracellular GAD antigens is not clear. However, it is interesting that SPS may also arise in individuals with autoantibodies to gephyrin, a cytosolic protein concentrated at the postsynaptic membrane of inhibitory synapses where it is associated with GABA_A _receptors [42]. This provides a further example of chronic rigidity and spasm possibly secondary to disruption of the inhibitory synapses.

Mutations in the *CLN3 *gene are thought to be responsible for the neurodegenerative disorder Batten disease (OMIM:204200). In *cln3*-knockout mice autoantibodies to GAD_65 _have been reported to be associated with brain tissue and result in inhibition of GAD activity [43]. These mice also demonstrate elevated brain glutamate levels as compared with controls, which may have a causative role in the astrocytic hypertrophy evident in *cln3*-knockout mice and along with anti-GAD_65 _autoantibodies in Batten disease patients may contribute to the associated preferential loss of GABAergic neurons.

Drugs, which potentiate the action of GABA, such as benzodiazepines and baclofen, ameliorate muscle rigidity and spasticity. These GABA agonists are thought to counter disinhibition of the velocity-dependent increase in skeletal muscle during stretch reflexes, observed in spasticity, which is the result of inadequate presynaptic inhibition of the muscle spindles [44,35,37]. γ-Vinyl-γ-aminobutyric acid (GVG) is used to treat neurological disorders including epilepsy, tardive dyskinesia and spasticity. It has been reported that it is the GABA-elevating effect of this compound that is responsible for its anti-convulsive properties [20].

We have identified a *GAD1 *sequence change G(36)C, which segregates with autosomal recessive spastic CP in 4 affected siblings. This nucleotide substitution causes a missense mutation, changing serine (12) to a cysteine in the *N*-terminal domain. This serine residue is conserved between all mammals (human/mouse/rabbit/pig) for which data are available. The association of GAD_67 _with membranes requires formation of heteromeric links with GAD_65_, which are mediated via their *N*-terminal domains. The *N*-terminus of GAD_65 _is palmitoylated and binds to the cellular membrane. The first 27 amino acids appear to be essential in this function [32]. The palmitoylation of cysteines 30 and 45 of GAD_65_, and the inability of residues 1–29 of GAD_67_, to substitute for this region, highlights the potential impact on cellular localisation of a nucleotide substitution in this domain [20]. GAD_65 _also undergoes phosphorylation of the first four serine residues in the *N*-terminal domain. These post-translational modifications highlight the importance of the flexibility and accessibility of this domain. *N*-terminal epitopes of GAD_65_, in the region corresponding to the residue, which undergoes mutation in GAD_67_, are particularly prominent autoantigens [45].

S(12)C amino acid substitution may thus produce subtle effects on cellular localisation, protein-protein interactions and/or protein processing, with a subsequent effect on GABA production. This is not inconsistent with the mouse *Gad1 *knockout where complete loss of *Gad1 *enzymatic function (~20% reduction of total Gad activity in the cerebral cortex) resulted in a cleft palate phenotype and neonatal death [28,30]. There is redundancy of GABA production as a result of the presence of two GAD proteins, and the precise function of each isoform may differ between man and mouse. It is interesting to note that the GAD_25 _splice variant of GAD_67_, also contains the S(12)C amino acid substitution in affected CP patients. This truncated variant is identical to the first 213 amino acids of GAD_67_, with the addition of an extra 11 *C*-terminal residues. It lacks the binding site for the cofactor PLP and is believed to lack any GAD activity [25]. The function of GAD_25 _is not known, but it may compete with GAD_67 _for incorporation into protein complexes. Therefore the presence of an *N*-terminal mutation would affect both GAD_25 _and GAD_67_, and may disrupt a complex regulatory mechanism for GAD_67_.

## Conclusions

This study illustrates the difficulty of gene cloning in rare autosomal recessive diseases mapped in small, consanguineous pedigrees. Identification of an ancestral haplotype allows refinement of the locus, but this has not been possible in this example. Within the minimal linkage region, any sequence change will segregate with the disease phenotype. Detection of a nonsense mutation leading to a protein truncation would provide compelling support for a mutation as causative. However, in the present example we have not seen such a mutation in the candidate gene so far examined. We are now expressing the variant forms of GAD_67 _(S12C) and GAD_25 _(S12C), as recombinant proteins, to assess catalytic activity and binding properties with respect to their normal counterparts. However, it may well prove difficult to detect subtle effects based on sub-cellular localisation changes in the mutant proteins, or changes in post-translational modification patterns.

Stability of the mRNA transcripts from these variants will be assessed by transfection studies in neuronal cells. Eventually, it would be of interest to attempt knock-in experiments with *GAD1 *(G36C), into the *Gad1-/- *mouse to see if this can rescue the cleft palate phenotype and reveal a CP-like picture. These experiments will be reported elsewhere. However, the possibility that reduced GAD_67 _activity may cause CP in the patients studied herein, in a manner reminiscent of that seen in SPS, leads us to report our findings at this stage. The success reported in treating SPS with intravenous immunoglobulin [40,46], suggests further evaluation of GABA agonists in the management of this difficult clinical problem.

## Abbreviations

BAC Bacterial artificial chromosome

BLAST Basic local alignment search tool

CEPH Centre d'Etude du Polymorphisme Humain

CHEF Contour-clamped homogenous electric field

CNS Central nervous system

CP Cerebral Palsy

CSF Cerebrospinal fluid

DNA Deoxyribonucleic acid

EST Expressed sequence tag

GABA Gamma-aminobutyric acid

GAD L-Glutamate decarboxylase

GVG Gamma-vinyl-gamma aminobutyric acid

HGMP Human genome mapping project

IDDM Type 1 Insulin-Dependent Diabetes Mellitus

MRI Magnetic resonance imaging

NCBI National Centre for Biotechnology Information

PAC Plasmid artificial chromosome

PCR Polymerase chain reaction

PDE Pyridoxine-dependent epilepsy

PFGE Pulse field gel electrophoresis

PLP Pyridoxal 5'-phosphate

PVL Periventricular leukomalacia

SPG Spastic paraplegia

SPS Stiff Person Syndrome

SSCP Single-strand conformational polymorphism

STS Sequence tagged site

YAC Yeast artificial chromosome

## Competing interests

The author(s) declare that they have no competing interests.

## Authors' contributions

CNL carried out the molecular genetic studies, sequencing and drafted the manuscript. The YAC mapping work was undertaken by JPL and CNL. AFM, DTB and IMC conceived the study and participated in the design and coordination, they also secured financial sponsorship from the Wellcome Trust and MRC. RA, SM, ERM and CGW recruited and gained consent from the families detailed in this study. All authors read and approved the final manuscript.

## Pre-publication history

The pre-publication history for this paper can be accessed here:


